# Evaluation of cooperative and non-cooperative game theoretic approaches for water allocation of transboundary rivers

**DOI:** 10.1038/s41598-022-07971-1

**Published:** 2022-03-07

**Authors:** Fahimeh Mirzaei-Nodoushan, Omid Bozorg-Haddad, Hugo A. Loáiciga

**Affiliations:** 1 Department of Irrigation & Reclamation Engineering, Faculty of Agricultural Engineering & Technology, College of Agriculture & Natural Resources, University of Tehran, Karaj, 3158777871 Iran; 2 Department of Geography, University of California, Santa Barbara, CA 93106 USA

**Keywords:** Climate sciences, Environmental sciences, Hydrology, Energy science and technology, Engineering, Mathematics and computing

## Abstract

Efficient water allocation in a transboundary river basin is a complex issue in water resources management. This work develops a framework for the allocation of transboundary river water between the countries located in the river basin to evaluate the characteristics of allocation approaches. The allocation of river water is obtained based on initial-water conditions, cooperative, and non-cooperative game-theoretic approaches. The initial-conditions water allocation approach assigns 34, 40, and 26% of the Harirud River flow to Afghanistan, Iran, and Turkmenistan, respectively. The game-theoretic cooperative approach assigns 36, 42, and 22% of the river flow to Afghanistan, Iran, and Turkmenistan, respectively. The non-cooperative game-theoretic approach establishes that the most stable water allocation was 42, 38, and 20% of the Harirud River flow for Afghanistan, Iran, and Turkmenistan, respectively. Human and agricultural water-stress criteria are used to evaluate the water allocations in the Harirud River basin. The criterion of human water stress has the largest influence in Iran, and the criterion of agricultural water stress has the smallest influence in Afghanistan. This work’s results indicate the initial-conditions water allocation approach favors Turkmenistan, whereas the cooperative and the non-cooperative game-theoretic approaches favors Iran and Afghanistan, respectively. The results show that the priorities of each country governs water allocation, and cooperation is shown to be necessary to achieve sustainable development.

## Introduction

Water management in transboundary river basins involves technical and scientific assessments, and legal, economic, cultural, social, and historical factors that cross political and cultural boundaries. Transboundary river management can improve food security, economic growth, adaptation to climate change, international trade, public health, and international cooperation (Mirzaei-Nodoushan et al.^1^). Management of transboundary river basins requires adaptive management which means the countries can adapt to changes and stresses of water resources and reduce their effect on sustainability (Wolf^2^).

Sustainable development is an effort to balance between economic evolutions, environmental issues, and social and welfare needs of a region in the present without damaging the future generations’ resources (Karimian et al.^3^). The challenges of the water crisis increase in shared water regions, and water managers must cooperate to manage water in a beneficial manner. Determining the benefits from the cooperation between countries and how they are distributed among the stakeholders is a nontrivial task in transboundary river basin management (Wolf^2^). To this end, it is necessary to increase the institutional capacity so that beneficial water management and water use can be established at all levels of society, and to ensure that governance organizations become active and effective in decision-making.

The allocation of water resources among transboundary stakeholders has been approached from various perspectives (Bozorg-Haddad et al.^4^). The most common solutions for water allocation are optimization methods and game theory approaches (Akbari-Alashti et al.^5^). As an example, Kazemi et al.^6^ reported a multi-objective optimization model for the allocation of water resources in the Sefidrood River Basin, Iran. The objectives of their model were to maximize revenue and minimize the Gini index. The results showed that the Gini index increases (an increase of injustice) with the increasing number of dams.

The genesis of game theory is traced to von Neumann and Morgenstern^7^, who provided a mathematical method for the analysis of human behavior in strategic decision-making by competitive or cooperative entities. Nash^8^ revolutionized game theory with the introduction of the Nash equilibrium for n-player games. The Nash equilibrium defines the solution of non-cooperative games. It occurs when each player knows the equilibrium strategies of the other players and no player has anything to gain by changing only his or her strategy (Holt and Roth^9^). Many decision-making situations involving the allocation of scarce resources under cooperative or non-cooperative behaviors can be solved with game theory (Harrison and List^10^), as done in this work for the allocation of a transboundary river’s water. Degefu et al.^11^ proposed a framework in which the theory of bargaining was combined with the allocation of resources and bankruptcy games to allocate water and improve welfare in the transboundary river basin. The Euphrates River was selected as the study area that encompasses parts of Turkey, Syria, and Iraq, and a framework was created for the economic redistribution of water and maximizing overall welfare. Arfanuzzaman and Abu Syed^12^ examined the relationship between ecosystem and water needs in the Teesta River Basin between India and Bangladesh using game theory. Their results showed that the zero-sum game perspective can be used to develop transboundary water policies. Zeng et al.^13^ applied a cooperative game theory method to solve transboundary water problems between two cities in the Guanting basin of northern China. The results demonstrated that full cooperation leads to the greatest total benefit to each stakeholder. Qin et al.^14^ proposed an integrated decision support framework combining multi-criteria decision making, bankruptcy theory, and the power index in the Dongjiang River Basin in China. The results showed that the proposed method is reliable for simultaneously considering the criteria of fairness and sustainability in the management of transboundary rivers.

The HBV model is a lumped conceptual catchment model that has relatively few model parameters and minimal forcing input requirements, usually the daily temperature and the daily precipitation (Bergström and Göran^15^). HBV stands for the Swedish title Hydrologiska Byråns Vattenbalansavdelning which was developed in 1976. The model is a popular rainfall-runoff model in simulating gauged and ungauged basins (Yang et al.^16^) that has been applied in different climate regions (Seibert and Beven^17^; Samuel et al.^18^; Pool et al.^19^).

Cooperative management of transboundary rivers contributes to sustainable water management and improves the chances of rendering benefits for all riparian countries (Mirzaei-Nodoushan et al.^1^). Yet, riparian countries, especially upstream ones in transboundary basins, are sometimes hesitant to be cooperative, and they tend to pursue self-serving use of river water. In this context, it seems necessary to consider strategies for water allocation according to realistic conditions within transboundary basins and to examine the effect of the strategies on transboundary countries. Past studies of water allocation lack a consistent framework to assess water allocation in transboundary basins.

This paper presents and analyses three approaches for transboundary water allocation that is displaying different decision-making of stakeholders. This study intends to show two types of common decision-making (behavior) of countries in water allocation and show the effect of these behaviors in meeting the water needs of countries that are not presented before in past studies. First is the allocation of water among competing users according to an initial-conditions water allocation of water that is made based on geographic location, economic development, and historical water use. The second approach involves game theory, wherein several coalitions of water users (or players in the game-theory jargon) are formed in which the players have specific functions. Coalition revenues are distributed among the players of a coalition based on inter-player cooperation and their agricultural, urban, industrial, and environmental water requirements. The third approach for transboundary water allocation is based on non-cooperative game theory. Finally, three approaches are evaluated based on the riparian countries’ characteristics.

## Methods

### The genetic algorithm (GA)

The GA is a pioneering evolutionary algorithm for optimization (Holland^20^). The GA and its variants mimic evolutionary processes in nature (mutation, cross-over, survival of the fittest) to improve an initially, randomly, generated population of possible solutions iteratively until it converges to a near-global solution. The reader is referred to Bozorg-Haddad et al.^21^ for a description of the GA and some of its variants with codes available for their programming.

### Mathematical programming model

This section presents a general mathematical programming model for a transboundary river system that serves urban, agricultural, and industrial water demands.

#### Objective functions

Two objective functions were implemented in this study. One maximizes the water supply reliability and was applied to obtain an initial-conditions water allocation. The second objective function maximizes the agricultural revenue by applying cooperative and non-cooperative game theory. Reliability measured the probability of a system performing its function satisfactorily. Volumetric reliability, specifically, was equal to the volume of water supply over the volume of water demand during the operational period. The equation for volumetric reliability (${\alpha }_{v}$) is as follows:

1 $${\alpha }_{v}=\frac{\sum_{t=1}^{N}{AW}_{t}}{\sum_{t=1}^{N}{De}_{t}},$$

where ${AW}_{t}$ = volume of water supplied at time *t,* and ${De}_{t}$ = volume of water demand at time *t*. ${AW}_{t}$ and ${De}_{t}$ were calculated as follows:

2 $${AW}_{t}=\sum_{k=1}^{K}\sum_{i=1}^{{I}_{k}}{(XA}_{i,k,t}+{XU}_{i,k,t}+{XD}_{i,k,t}),$$

for all $t$, where ${XA}_{i,k,t}$, ${XU}_{i,k,t}$, and ${XD}_{i,k,t}$ denoted the volumes of water used by the agricultural, urban, industrial sectors at location $i$ in country *k* and time *t,* respectively. The agricultural water use at location $i$, country $k$, and time $t$ are given by:

3 $${XA}_{i,k,t}=\sum_{j=1}^{{J}_{k}}{Ar}_{i,j,k,t} \times I{r}_{i,j,k},$$

for all $i$, $k$, $t$, where ${Ar}_{i,j,k,t}$, $I{r}_{i,j,k,}$ = area of crop *j* at location $i$ in country *k* and time $t$, and consumptive (water) use of crop *j* (volume of water per area) at location $i$ in country $k$, respectively. The volume of water demand at time *t* is calculated with Eq. (4):

4 $${De}_{t}=\sum_{k=1}^{K}\sum_{i=1}^{{I}_{k}}\left({DeA}_{i,k,t}+{DeU}_{i,k,t}+{DeD}_{i,k,t}\right),$$

for all *t,* where ${DeA}_{i,k,t}$, ${DeU}_{i,k,t}$, ${DeD}_{i,k,t}$ denote the agricultural, urban, and industrial sectors’ water demands or needs, respectively, at location *i*, country *k*, and time *t.*

The agricultural revenue is calculated as follows:

5 $${B}_{agr}=\sum_{t}\sum_{k}\sum_{i}\sum_{j}{\mathit{Pr}}_{j,k}\times {Ar}_{i,j,k,t}\times {Yd}_{i,j,k },$$

where $${B}_{agr}$$, $${Pr}_{j,k}$$, and $${Yd}_{i,j,k}$$ = the agricultural revenue, price (say, in $/ton of produce) of crop *j* in country *k*, and the yield per unit area (say, in ton/hectare) of crop *j* at location $$i$$ in country *k*, respectively. The revenue to country *k* = *r, *$$1\le r\le K$$*,* is calculated with Eq. (5) by summing over *t*, *i, j* while setting the country index constant and equal to $$r$$. The decision variables of the mathematical programing problem are the areas $${Ar}_{i,j,k,t}$$ and the urban ($${XU}_{i,k,t}$$) and ($${XD}_{i,k,t}$$) industrial water uses.

#### Constraints

Water balance at each location $i$ of country $k$ and time $t$:

6 $${Qout}_{k,i,t}={Qin}_{k,i,t}-{XA}_{i,k,t}-{XU}_{i,k,t}-{XD}_{i,k,t},$$

for all $i, k, t$, where ${Qout}_{k,i,t}$ = the volume of water leaving location* i* in country *k* at time *t*, ${Qin}_{k,i,t}$ = the volume of water entering location* i* in country *k* at time *t*.

The water used by the agricultural, urban, and industrial sectors may not exceed their water demands. The constraints on water use are as follows:

7 $${XA}_{i,k,t}\le {DeA}_{i,k,t},$$

8 $${XU}_{i,k,t}\le {DeU}_{i,k,t},$$

9 $${XD}_{i,k,t}\le {DeD}_{i,k,t},$$

for all $i, k, t$. The nonnegativity constraints on water use and outflow are as follows:

10 $${{XA}_{i,k,t}, XU}_{i,k,t}, {XD}_{i,k,t}\ge 0,$$

11 $${Qout}_{k,i,t}\ge 0,$$

for all $$i, k, t$$.

### Game theory

Two types of game theory for decision making, cooperative and non-cooperative game theory, are described in this section. The countries sharing the transboundary river are modeled as players of a competitive game vying to maximize their payoffs, which in this case means maximizing the reliability of their water supply and their agricultural revenues.

#### Cooperative decision making

Each player is in this instance is primarily interested in the magnitude of his or her revenue from participating in a cooperative game rather than in increasing the total income of the cooperative game compared to what could be obtained by participating in a non-cooperative game. In a cooperative game players interact with each other before the game and they may sign contracts with each other and form alliances. The revenue from all possible strategies is expressed in the form of characteristic functions, such as revenue received from a game. A fair revenue sharing by cooperative players can be obtained with the Shapley value method (Shapley^22^).

#### Non-cooperative decision making

It is assumed in non-cooperative games that each player acts rationally in choosing his or her strategy. The choice of strategies in this type of game aims at maximizing the individual interests of each player. The concept of the Nash equilibrium is central to non-cooperative game theory. Nash equilibrium assumes that each player can only have one strategy during the game, and each player considers the strategies of the other players when making decisions. The Nash equilibrium may fail to accurately predict the interaction between players in reality. For this reason, other methods are used to improve the modeling of non-cooperative games in which each player attempts to anticipate the decisions of his or her rivals when choosing a strategy.

### Criteria for evaluating transboundary water management

Various political, social, economic, and environmental criteria are defined to determine the status of transboundary water resources. The United Nations Environment Programme (UNEP) has set out specific criteria for assessing the management of transboundary basins in the form of a codified approach (Hooper and Lloyd^23^). This study considers the human water stress and the agricultural water stress criteria, which are defined next.

#### Human water-stress criterion

The human water stress proposed by the United Nations is measured by the amount of water available per person per year. This criterion was obtained by dividing the amount of available water by the population served:

12 $$HI=\frac{AW}{{T}_{pop}},$$

where *HI* = human water-stress criterion, *AW* = amount of available water and *Tpop* = population served. The lower the value of this criterion, the higher the human water stress.

#### Agricultural water stress criterion

The agricultural water-stress criterion is equal to the amount of water available in a basin divided by the area of agricultural land:

13 $$AI=\frac{AW}{Ar},$$

where *AI*, *AW*, and *Ar* denote the agricultural water stress index, the volume of water available and the area of agricultural land respectively. Thus, the lower the value of this criterion, the higher the agricultural water stress.

**Figure 1:**
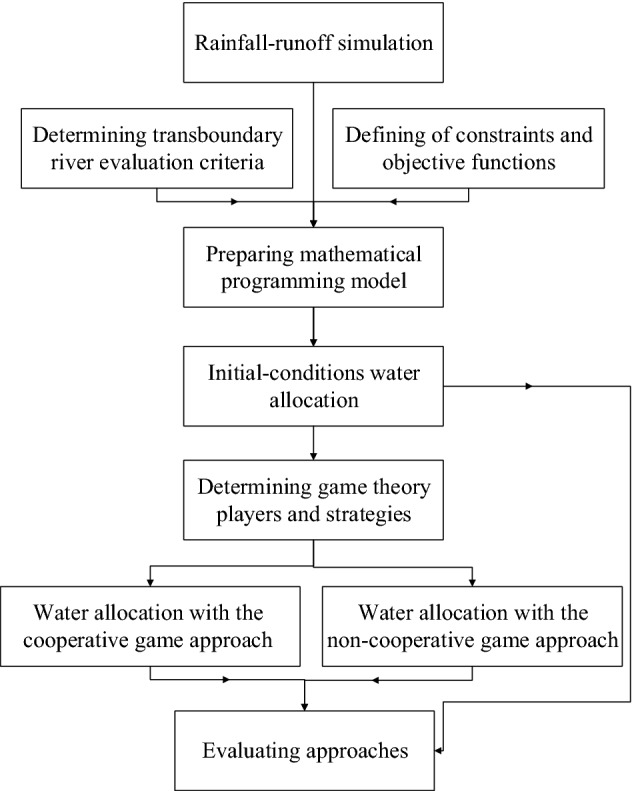
Methodology flowchart.

This paper’s methodology is displayed as a flowchart in Fig. 1, where it is seen that the river runoff was first obtained with the HBV rainfall-runoff model. The mathematical model given by Eqs. (1)–(11) was developed. The water allocation was obtained with the initial-conditions approach, and the cooperative and non-cooperative game theory approaches were then calculated. Lastly, the water allocations were evaluated with the human water-stress and the agricultural water-stress criteria.

### Study area

**Figure 2:**
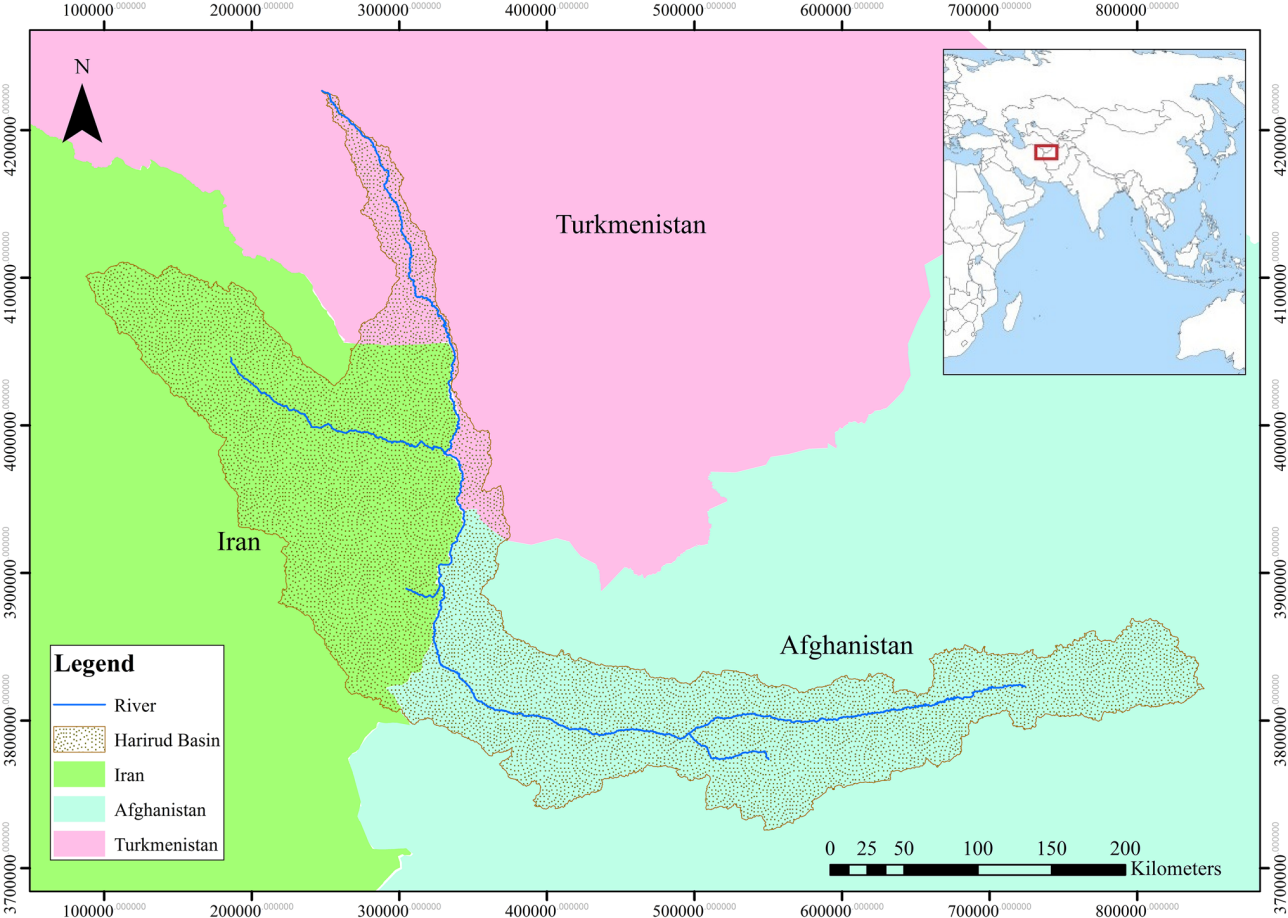
Geographical location of the Harirud river basin (ArcGIS, version: 10.5.0.6491, site: www.esri.com).

The Harirud River is a transboundary river in eastern Iran that flows through Afghanistan, Iran, and Turkmenistan and has been a source of water for all three countries since ancient times. Much of the river’s water flow, which originates in the highlands of Afghanistan, is controlled by the latter country through dams, leading to a significant reduction in water flow in the downstream countries (see Fig. 2).

### Ethics approval

All authors accept all ethical approvals.

### Consent to participate

All authors consent to participate.

### Consent for publication

All authors consent to publish.

## Results

There was lack of data in the Harirud Basin outside Iran and minimal data sharing between the transboundary countries. It was therefore necessary to apply rainfall-runoff simulation to make up for the data shortages. Rainfall-runoff simulations for the Harirud River basin were performed using the HBV conceptual model. Data on precipitation, temperature, potential evapotranspiration, and river flow for a 10-year (1969 through 1978) were implemented for model calibration. The observational time series of monthly river flow corresponded to the Robat Akhund gaging station in Afghanistan. The calibrated HBV model simulated monthly river flow from 1979 through 2014. The correlation coefficient (*R*^2^) of the simulated flows equaled 0.821, which indicated acceptable predictive skill or accuracy. The river water was allocated in this study based on the water demands within the Harirud River basin for the period 2000–2014.

### Initial-conditions water allocation

**Figure 3:**
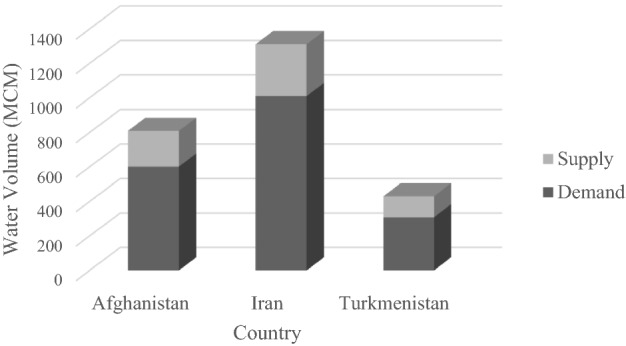
The average annual volume of water supply and demand for each country calculated with the initial-conditions water allocation approach. (MCM = 10^6^ m^3^).

The initial-conditions water allocation was calculated with the GA by maximizing the reliability of water supply. Water is allocated to the countries according to their water needs, and there are no constraints concerning cooperation to maximize the sum of agricultural revenue nor on non-cooperating countries and their behavior effects with respect to received water. This means the only constraints are those introduced in the mathematical programming model, and the objective function is to maximize the reliability of water supply. The average annual volumes of water supply and demand for each country calculated with the initial-conditions allocation are depicted in Fig. 3, where it is that the total water demands of countries are much larger than the available water. The water is allocated to the countries according to their demands so that the percentages of river flow allocated to Afghanistan, Iran, and Turkmenistan are on average equal to 34, 40, and 26, respectively.

### Water allocation with the cooperative game approach

The mathematical programming problem of water allocation maximizes the agricultural revenue. The priority of water withdrawal by the upstream country (Afghanistan) is considered because of its geographically advantageous position. The players in the coalition of cooperators must agree on revenue sharing. This sharing was obtained with the Shapley value method that establishes the relative contribution of each player to the revenue of the coalition (Shapley^22^). The contributions are listed in Table 1, where it is seen that Iran’s contribution to the coalition’s revenue is the highest and Turkmenistan’s contribution is the lowest. Iran earns more agricultural revenue due to its larger water demand and higher crop yields.

**Table 1:** Shapley value of countries.

Country	Shapley value (M$ = 10^6^$)
Afghanistan	13.23
Iran	27.63
Turkmenistan	6.37

A key issue in a cooperative game concerns the stability of a coalition, which can be evaluated with the Gately index. This index measures a player’s ability to harm other players if it leaves the coalition (Gately^24^). The calculated Gately index is shown in Table 2, where it was seen that Turkmenistan’s index is higher than those of the other countries. This implies that Turkmenistan is the most eager to leave the coalition. The Gately index establishes that Turkmenistan would benefit more if it only partners with Afghanistan or Iran. On the other hand, the value of this index for Iran is less than those of the other countries, which means that it would cause more harm to itself than to the other countries if it leaves the coalition.

**Table 2:** Gately index of countries.

Country	Gately index
Afghanistan	1.30
Iran	1.14
Turkmenistan	9.39

**Figure 4:**
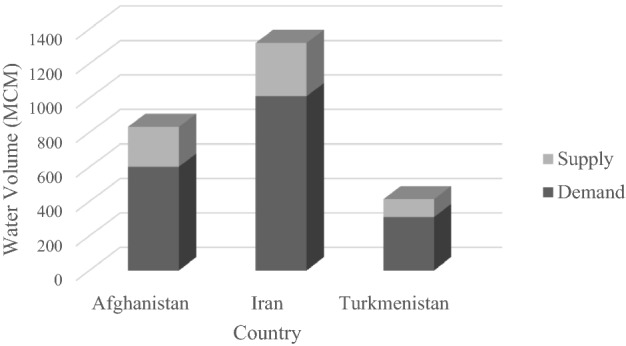
Average annual volume of water supply and demand calculated with the non-cooperative allocation approach (MCM = 10^6^ m^3^).

The water allocations obtained with the cooperative approach are displayed in Fig. 4. The water allocations to Afghanistan, Iran, and Turkmenistan equalled 36%, 42%, and 22%, respectively, of the river flow. This implies that Afghanistan’s and Iran’s shares of river water increases and Turkmenistan’s share decreases in comparison to the initial-conditions water allocation. This approach dictates that a country with higher crop yields and wider arable land acquires a larger share of the available water. The upstream power of water withdrawal by Afghanistan means that this country enjoys a significant volume of water, despite having fewer inhabitants and less arable land than Iran. Also, Turkmenistan’s water share decreases because of its lower crop yields and having less arable land than Iran, which affects the objective function maximizing agricultural revenue.

### Water allocation with the non-cooperative game approach

Each country devises strategies for interacting with the other countries. Afghanistan occupies the upstream portion of the Harirud Basin, and it may pursue a compromise strategy and allow larger flow to the downstream countries, resulting in larger revenue for Iran and Turkmenistan. Afghanistan, on the other hand, may use more water and reduce the downstream revenue. The downstream countries have two strategies: (1) they can choose a strategy of compromise with Afghanistan’s strategy seeking to improve basin management, or (2) they can adopt a strategy of opposition to Afghanistan’s strategy and seek to undermine it.

The formulation of the non-cooperative game is adjusted by incentives from the downstream countries to the upstream county, and the optimization is implemented to maximize agricultural revenue. The incentive is structured such that Iran and Turkmenistan would give Afghanistan $1.5 million and $0.5 million, respectively, for each additional million cubic meters of water that Afghanistan releases to the downstream countries.

The combinations of strategies of the three countries are listed in the payoff matrix of Table 3. The values of the payoff matrix represent the annual revenue accruing from the use of the Harirud River water received by Afghanistan, Iran, and Turkmenistan. The payoff to each country specified by the payoff matrix depend on its strategies (compromise or opposition) and the combinations of the other countries’ strategies. The strategy corresponding to each situation yielding the highest payoff constitutes the Nash equilibrium of that country corresponding to that situation. Examining the payoff matrix reveals that the only situation in which the three countries reach a state of equilibrium when Afghanistan has an oppositional strategy and Iran and Afghanistan have compromising strategies.

**Table 3:** Country payoff matrix corresponding to the countries’ strategies (annual revenue M$ = 10^6^$).

Afghanistan	Iran	Turkmenistan					
		Compromise			Opposition		
Compromise	Compromise	44.71	63.60	12.34	45.51	**65.67**	**12.98**
	Opposition	47.03	**63.76**	**12.68**	46.29	64.80	11.74
Opposition	Compromise	**46.89**	**64.70**	**13.52**	**49.93**	**66.15**	11.79
	Opposition	**48.10**	60.91	**13.09**	**48.30**	61.15	10.08

Significance values are given in bold.

**Figure 5:**
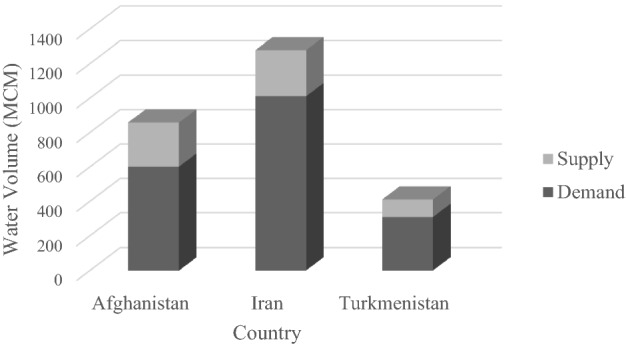
Average annual volume of water supply and demand calculated with the non-cooperative allocation approach (MCM = 10^6^ m^3^).

The annual water allocation calculated with the non-cooperative approach when Afghanistan is oppositional and Iran–Turkmenistan compromise is depicted in Fig. 5. In this situation the Nash equilibrium is of a non-cooperative nature. The water allocations to Afghanistan, Iran, and Turkmenistan represent 42, 38, and 20% of the river flow, respectively. In this instance Afghanistan’s share of river water is the largest, and Iran’s and Turkmenistan’s shares are the lowest in comparison to the other allocations. The results obtained in this approach are closer to the current state of water allocation in this basin. Yet, Iran has a lower share of water, and Turkmenistan has a larger share of water in the current state of water allocation than the shares prevailing in the non-cooperative allocation approach. The water released downstream by Afghanistan featuring an oppositional stance is divided equally between Iran and Turkmenistan.

### Analyzing the results of the calculated allocations

**Figure 6:**
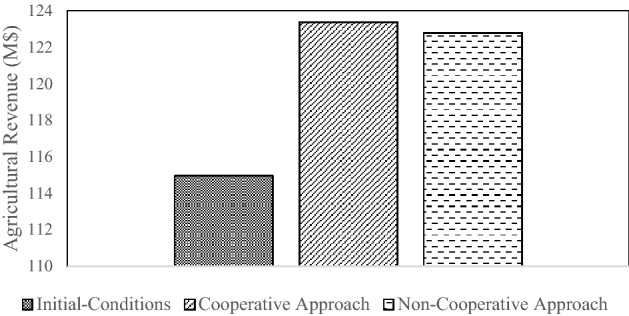
Total annual revenue calculated with the three allocation approaches (M$ = 10^6^$).

The combined annual revenues of the countries corresponding to the three allocation approaches are depicted in Fig. 6. The largest agricultural combined revenue for Afghanistan, Iran, and Turkmenistan is obtained with the cooperative approach, and the initial-conditions water allocation produces the smallest revenue. Turkmenistan has a larger share of water and less value-added agriculture according to the initial-conditions water allocation approach. In this instance the total annual agricultural revenue of the countries is reduced. Iran’s share of river water increases when there is cooperative sharing compared to the other two approaches (i.e., initial-conditions and non-cooperative), and agricultural revenues are largest due to the high value-added of Iran’s agriculture compared to those of other countries.

**Figure 7:**
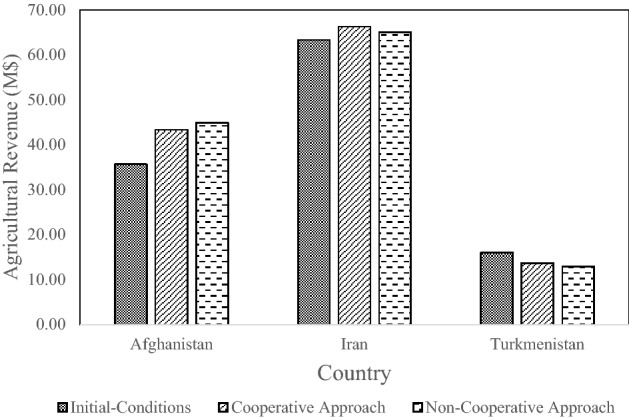
Annual agricultural revenue of each country calculated with the three allocation approaches (M$ = 10^6^$).

The individual countries’ revenues are displayed in Fig. 7, where it is seen that the largest revenue for Afghanistan, Iran, and Turkmenistan corresponds to the non-cooperative, cooperative, and initial-conditions allocations, respectively. It pays for Afghanistan to be oppositional because the Nash equilibrium is achieved when its strategy is oppositional and the other two countries compromise. Therefore, Afghanistan does not release excess water downstream, but the two downstream countries compromise and provide incentives to Afghanistan to release water. The two downstream countries act in a compromising manner so that the amount of water they receive is not reduced.

**Figure 8:**
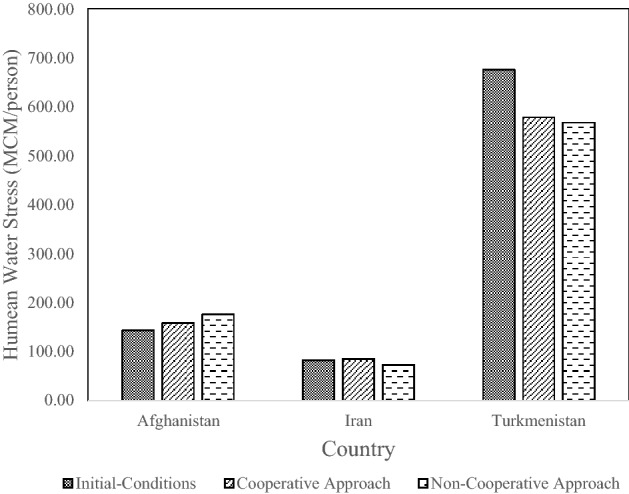
Human water stress criteria for each country (MCM = 10^6^ m^3^).

Figure 8 shows the results associated with the human water-stress criterion. Turkmenistan endures the lowest tension (i.e., the largest value of this criterion) because it has a smaller population than the other countries. This means that the amount of water allocated to each person was relatively high, especially that obtained with the initial-conditions water allocation approach. Iran had the smallest value of the human water stress and this means it endures the highest tension, i.e., the smallest volume of water per capita due to having the largest population living in the basin.

**Figure 9:**
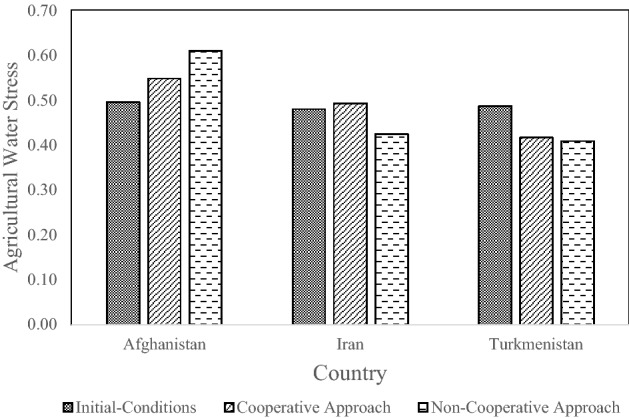
Agricultural stress criteria for each country.

Figure 9 depicts the results obtained for the agricultural water-stress criterion. Afghanistan features the most favorable value of this criterion, especially for the non-cooperative approach. In other words, the volume of water allocated per unit of agricultural land in Afghanistan is the largest.

It is concluded from the data presented in Figs. 7, 8, and 9 that Afghanistan receives the largest agricultural revenue, the lowest water stresses with respect to human water supply and agricultural water supply under the non-cooperative approach. It is true that the cooperative approach produces the largest total agricultural revenue (Fig. 6). At the same time, the benefits of each country demonstrate that all countries receive less benefits under this approach. It is obvious that available water is constant and if one country takes more water from the river another country must take less. Therefore, it is fair to state that no approach is better than others because each country chooses its preferred approach based on its benefits. The actual situation in the Harirud basin is that the upstream country enjoys the largest water withdrawal, while Iran and Turkmenistan have adopted compromise strategies in the hope of changing Afghanistan’s strategy.

## Conclusion

This study developed a mathematical programming model for water allocation to maximize the interests of the countries in a transboundary river basin and apply restrictions on their uses of the transboundary river water. The river system features one upstream country and two downstream countries. The volumes of water available to meet the countries’ urban and agricultural water demands are considered in this study.

The GA was applied to allocate the transboundary river water. The initial-conditions water allocation was performed by maximizing water supply reliability. The percentages of the Harirud River’s flow allocated to Afghanistan, Iran, and Turkmenistan equalled 34, 40, and 26, respectively.

Water allocation was carried out with a cooperative game theory approach to maximize agricultural revenue. The shares of river flow allocated to Afghanistan, Iran, and Turkmenistan were 36, 42, and 22%, respectively. Turkmenistan was the most likely country to leave the coalition. Iran's Shapley value was higher than those of the other countries in the coalition because its contribution to the coalition’s revenue profits is the largest by virtue of its large agricultural output.

River water allocation was also calculated with a non-cooperative game theory approach. Countries could choose to be compromising or oppositional. Afghanistan releases more water downstream when taking the compromise strategy, and increases the amount of water withdrawn for itself when it chooses to be oppositional. Iran and Turkmenistan pay incentives to lure Afghanistan to be compromising and deprive Afghanistan of this benefit if it chooses to be oppositional.

The Nash equilibrium outcome of each country was determined by entering the results obtained from the combination of the countries’ strategies in the payoff matrix. The Nash equilibrium in this matrix occurs when Afghanistan is oppositional and the other two countries are compromising. This situation gives rise to a Nash equilibrium under non-cooperation in which the water allocations to Afghanistan, Iran, and Turkmenistan are 42, 38, and 20% of the river’s flow, respectively.

The allocations were evaluated using stress criteria. The human water-stress criterion indicates that Iran, due to having the largest population in the Harirud River basin, endures stress. Instead, Turkmenistan endures the least stress. The agricultural stress criterion was also calculated by the three water-allocation approaches, and the results showed that the amount of water allocated per unit of agricultural land in Afghanistan is the largest. Iran and Turkmenistan face relatively similar agricultural stress. The results concerning the evaluation criteria indicate the best revenues for Turkmenistan, Iran, and Afghanistan are obtained with the initial-conditions water allocation, the cooperative, and the non-cooperative approaches respectively.

This study’s results demonstrate that transboundary river water must consider all probable strategies and all possible approaches. In this manner, countries can strategize based on the benefits and costs associated with probable strategies. The results obtained for the Harirud Basin explain the strategies followed by Afghanistan, Iran, and Turkmenistan in recent years. Afghanistan earns the most revenues from water use dictated by a non-cooperative approach. Iran attempts to meet its water needs with a cooperative approach. Turkmenistan is satisfied with the current situation and cooperates with Iran because it currently receives 50% of the water downstream (more than the share it would receive if it follows different strategies).

This study showed that the allocation results for water allocation of a transboundary river basin will change depending on the variable behavior of countries and the uncertainty in the decisions of countries located in the basin. Results denoted that the upstream country is not willing to cooperate because of the benefits of maximum consumption. Also, the downstream country, which is larger in terms of agricultural economy and number of inhabitants in the region and irrigable land area, tends to cooperate with the upstream country in order to meet the needs of its region. In other words, for further research, it is necessary to add other parameters that are benefits other than the benefits of water consumption to the issue of allocation so that all countries may become cooperative. In the current situation, the Harirud basin does not have sustainable management of water resources and the environment, and probably will face more serious problems in the future for all riparian countries. Cooperative management of the transboundary river basin would produce the best long-term outcomes for the transboundary countries. This would require the countries to make some concessions in exchange for achieving sustainable water management that would produce benefits for all of them indefinitely.

## Data Availability

The data that support the findings of this study are available from the corresponding author upon reasonable request.

## Code availability

The codes that support the findings of this study are available from the corresponding author upon reasonable request.
